# Molecular Imaging of Acute Graft-Versus-Host Disease

**DOI:** 10.2967/jnumed.123.266552

**Published:** 2024-03

**Authors:** Chiara Bernardi, Valentina Garibotto, Behnaz Mobashwera, Robert S. Negrin, Israt S. Alam, Federico Simonetta

**Affiliations:** 1 Division of Hematology, Department of Oncology, Geneva University Hospitals, Geneva, Switzerland;; 2 Translational Research Center for Oncohematology, Department of Medicine, University of Geneva, Geneva, Switzerland;; 3 Division of Nuclear Medicine and Molecular Imaging, Geneva University Hospitals, Geneva, Switzerland;; 4 CIBM Center for Biomedical Imaging, Geneva, Switzerland;; 5 Department of Hematology, Southampton General Hospital, University Hospital Southampton, Southampton, United Kingdom;; 6 Division of Blood and Marrow Transplantation and Cellular Therapy, Stanford University, Stanford, California; and; 7 Molecular Imaging Program at Stanford, Department of Radiology, Stanford University School of Medicine, Stanford, California

**Keywords:** hematology, molecular imaging, PET/CT, graft-versus-host disease, clinical, preclinical

## Abstract

Noninvasive molecular imaging of acute graft-versus-host disease (GvHD) after allogeneic hematopoietic stem cell transplantation has great potential to detect GvHD at the early stages, aid in grading of the disease, monitor treatment response, and guide therapeutic decisions. Although the specificity of currently available tracers appears insufficient for clinical GvHD diagnosis, recently, several preclinical studies have identified promising new imaging agents targeting one or more biologic processes involved in GvHD pathogenesis, ranging from T-cell activation to tissue damage. In this review, we summarize the different approaches reported to date for noninvasive detection of GvHD using molecular imaging with a specific focus on the use of PET. We discuss possible applications of molecular imaging for the detection of GvHD in the clinical setting, as well as some of the predictable challenges that are faced during clinical translation of these approaches.

Allogeneic hematopoietic stem cell transplantation (HSCT) is a potentially curative therapy for a broad range of hematologic diseases. Unfortunately, allogeneic HSCT is still associated with significant morbidity and mortality related to transplant complications, namely acute graft-versus-host disease (GvHD). During acute GvHD, donor-derived T cells interact with host tissues, leading to their activation, proliferation, and migration to target organs, notably skin, liver, and intestine (1). Current approaches for the diagnosis of acute GvHD are based on clinical and pathologic elements that are restricted to later, symptomatic, stages of the disease. Given the importance of timely therapeutic interventions for acute GvHD treatment, the use of noninvasive imaging modalities to predict and detect GvHD early, prior to symptom onset, could greatly benefit patient outcomes. Conventional radiology using ultrasound, contrast-enhanced CT, or MRI is of limited utility for acute GvHD diagnosis as any morphologic changes are often nonspecific (2).

Molecular imaging allows noninvasive measurements of biologic processes at the cellular and subcellular levels, and its application to the study of the immune system is a rapidly expanding field. PET imaging is a highly sensitive and quantitative clinical molecular imaging modality, perfectly poised to provide noninvasive, whole-body mechanistic insights into disease pathogenesis. The use of targeted PET probes specifically allowing detection of cellular or molecular processes involved in GvHD pathogenesis thus represents a promising approach for early detection of acute GvHD at presymptomatic stages.

According to the classic model, acute GvHD pathogenesis can be divided into 3 phases (Fig. 1): host tissue damage resulting from the conditioning chemotherapy; activation of donor and recipient antigen-presenting cells and subsequent donor T-cell activation and expansion; and an effector phase in which activated donor T cells cause tissue damage by targeting host cells, inducing apoptosis. Using current tools, acute GvHD is diagnosed predominantly during the third phase on the basis of a combination of clinical symptoms and histologic findings. Major efforts have been undertaken to identify biomarkers to predict and diagnose GvHD at earlier stages. Molecular imaging approaches have the potential to allow diagnosis as early as the second phase, before overt signs of GvHD (Fig. 1). Imaging agents targeting metabolic pathways have the potential to report on all 3 phases of GvHD, whereas the use of small molecules or monoclonal antibodies (mAbs) to image T cells could help elucidate phase 2 dynamics. Finally, imaging agents evaluating tissue damage may allow detection and monitoring of immunopathologic processes resulting from phase 3 of GvHD pathogenesis. In this review, we summarize the clinical and preclinical molecular imaging technologies available for acute GvHD diagnosis and monitoring and discuss future prospects for clinical translation.

**FIGURE 1.**
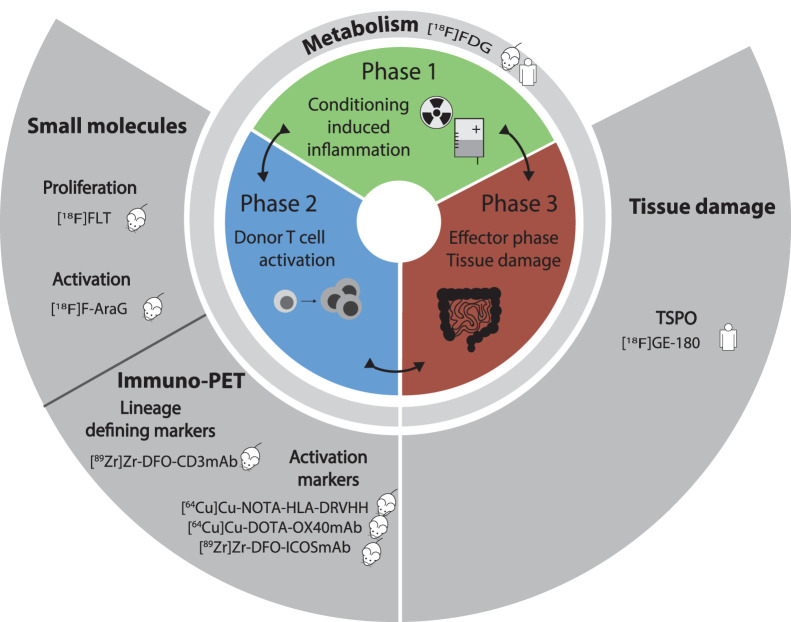
Summary of preclinical (mouse icon) or clinical (human icon) studies to image different phases of acute GvHD pathogenesis. VHH = variable fragments of heavy chain antibody.

## IMAGING METABOLISM

### ^18^Fluorine-fluorodeoxyglucose ([^18^F]FDG)

**FIGURE 2.**
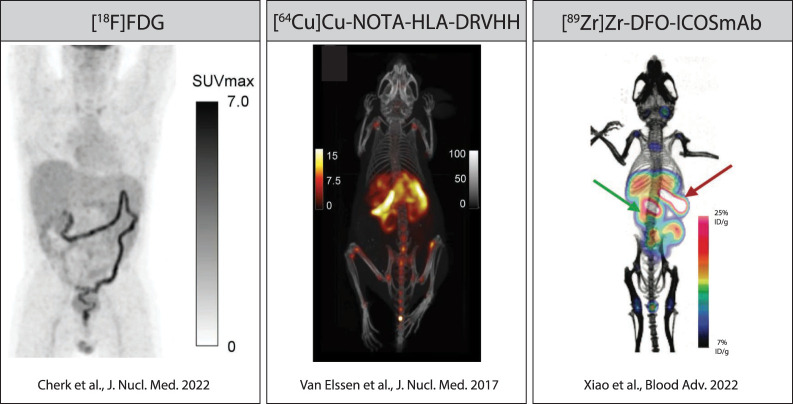
Examples of preclinical and clinical PET-imaging approaches to imaging acute GvHD. In Cherk et al. (4), patients received [^18^F]FDG (3 MBq/kg) and were imaged 60–80 min later. In Van Elssen et al. (11), mice were given 1.85 MBq (∼5 μg) of [^64^Cu]Cu-DOTA-OX40mAb and were imaged 2 h after injection. In Xiao et al. (16), mice received 1.85 MBq (∼7 μg) of [^89^Zr]Zr-DFO-ICOSmAb and were imaged 48 h later. VHH = variable fragments of heavy chain antibody. (Reprinted from (4*,*^11^*,*^16^).)

[^18^F]FDG, the most widely used PET tracer in clinical practice, takes advantage of the higher glucose consumption by metabolically active tissues. Given the wide range of metabolically active cells and tissues, [^18^F]FDG is thought to annotate all 3 phases of GvHD pathogenesis (Fig. 1). Early reports suggested that [^18^F]FDG PET can visualize tissue inflammation associated with acute gastrointestinal GvHD (GI-GvHD). In a retrospective analysis conducted on 101 patients with suspected acute GI-GvHD, 74 of whom were clinically or histologically proven to have acute GI-GvHD, [^18^F]FDG PET had a sensitivity of 93% and specificity of 73% (3). Moreover, SUV_max_ discriminated between patients with a fast or slow/no response to immunosuppressive therapies. False-positive cases were related mostly to intestinal infections. In a more recent prospective study, 51 allogeneic HSCT recipients with clinically suspected acute GI-GvHD underwent PET/CT followed by endoscopy and histologic analysis (Fig. 2, left panel) (4). Twenty-three patients had histologically proven upper or lower acute GI-GvHD. [^18^F]FDG PET was not able to distinguish between acute GvHD and non-GvHD inflammatory changes in the colon, yielding a sensitivity of 69%, a specificity of 57%, a negative predictive value of 73%, and a positive predictive value of 59%. To increase sensitivity and specificity, a pilot study on 21 patients with acute GI-GvHD used [^18^F]FDG PET/MRI (5). The acute GI-GvHD detection rate increased from 57% of [^18^F]FDG PET alone or 61% of MRI alone to 100% for [^18^F]FDG PET/MRI.

Collectively, these prospective and retrospective studies show promise that [^18^F]FDG PET/CT will have a role in GvHD diagnosis, but additional investigations are needed to evaluate the impact of the limited specificity of [^18^F]FDG in this indication.

## IMAGING T-CELL RESPONSES

Given the importance of T cells in GvHD pathogenesis, molecular imaging targeting T cells would be ideal for GvHD detection and monitoring, allowing imaging of the second, and to some extent the third, phase of GvHD (Fig. 1). Strategies to track T cells in vivo using molecular imaging have been reviewed extensively (6*,*^7^). The simplest approach, ex vivo radiolabeling of T cells before infusion, is limited by the short time frame in which analysis can be performed, which is due both to radioisotope decay and to tracer dilution caused by cell proliferation. Using such a strategy for GvHD would be challenging given the variable time frame in which GvHD develops after transplantation. T-cell–specific tracers will be likely required.

### Small Molecules

#### ^18^F-3′-Deoxy-3′-Fluorothymidine ([^18^F]FLT)

Given the high proliferation rate of T cells during GvHD, the use of [^18^F]FLT PET imaging has been attempted in murine models of allogeneic HSCT (8). FLT is a thymidine analog that is incorporated into the DNA at the time of replication and therefore reflects cellular proliferation. In murine models of GvHD, [^18^F]FLT allowed differentiation of control mice from mice that developed GvHD after receiving alloreactive T cells, by detecting higher tracer uptake in the lymph nodes and spleen of the latter. However, tracer uptake in GvHD-target organs, mainly the gastrointestinal tract, did not differ between GvHD and control mice because of high variability. This potentially represents a major limitation for the use of [^18^F]FLT for GvHD diagnosis given that signal outside target organs can originate from proliferation of hematopoietic cells other than T cells during engraftment. An early phase I study is ongoing and will show whether [^18^F]FLT uptake can predict GvHD development in patients who underwent HSCT (NCT03546556).

#### 2′-Deoxy-2′-[^18^F]Fluoro-9-β-D-Arabinofuranosyl Guanine ([^18^F]F-AraG)

AraG is the water-soluble prodrug of nelarabine, a drug known for its specific cytotoxicity toward T cells and clinically used in T-cell malignancies. AraG enters cells using nucleoside transporters and is phosphorylated by either cytosolic deoxycytidine kinase or mitochondrial deoxyguanosine kinase. At high doses, phosphorylated AraG induces T-cell death by inhibiting DNA synthesis at the low picomolar mass levels required for imaging, AraG specifically accumulates in T cells without inducing detectable cell death. Ronald et al. evaluated [^18^F]F-AraG in a mouse model of GvHD (9) and showed that this small molecule is able to visualize T-cell expansion in secondary lymphoid organs during GvHD. Unfortunately, the tracer’s high background hepatic signal precluded analysis of both the gastrointestinal tract and liver itself, 2 major GvHD-target organs. Given the spatial resolution and the favorable kinetics observed with [^18^F]F-AraG in humans, clinical evaluation of this approach for GvHD diagnosis was explored in a limited number of subjects (NCT03367962), although the trial was closed because of challenges in patient recruitment and selection.

### Immuno-PET

Immuno-PET exploits the high specificity of mAbs to selectively bind cells expressing the target antigen; radiolabeling these moieties with PET isotopes allows for in vivo visualization of those targeted cells. This technique is rapidly gaining traction as an approach to monitoring T cells without the need for their ex vivo manipulation. Most T-cell–targeting immuno-PET tracers developed so far can be classified on the basis of the antigen they target and fall into 2 major categories: tracers targeting T-cell lineage-defining molecules, (e.g., CD3, CD4, and CD8) and those targeting T-cell activation makers (e.g., HLA-DR [human leukocyte antigen–DR isotype], CD69, OX40 [CD134], 41BB [CD137], and inducible T-cell costimulator [ICOS]).

#### Immuno-PET Targeting T-Cell Lineage Markers

Targeting T-cell lineage-defining markers such as CD3, CD4, or CD8 is an obvious approach to immuno-PET imaging of T-cell–mediated processes, including GvHD. Given the importance of T-cell expansion in GvHD pathogenesis, namely during phase 2, the specific quantification of T-cell numbers at the target-tissue level and, moreover, the dynamic quantification of T-cell burden over time have great potential for GvHD diagnosis. The most clinically advanced T-cell–specific tracers to date are those targeting CD8, which have yet to be evaluated in the context of GvHD. To date, only CD3 immuno-PET has been reported in murine models of GvHD.

Pektor et al. used PET/MRI with a ^89^Zr (half-life, 78.4 h)-labeled antihuman CD3 mAb in a murine model of xenogeneic GvHD (10). The tracer exhibited higher uptake in GvHD-target organs, namely the liver, as well as in secondary lymphoid organs at different time points after peripheral blood mononuclear cell administration into lymphodepleted mice. Interestingly, the authors administered regulatory T cells as GvHD prophylaxis; this approach significantly reduced T-cell infiltration in regulatory T-cell–treated mice, visualized with CD3 immuno-PET. Although promising, this proof-of-concept report did not address one of the major risks of targeting CD3 in immunopathogenic contexts such as GvHD: that of potentially interfering with T-cell biology and exacerbating the disease. Targeting CD3 could also induce chronic T-cell stimulation and eventually lead to T-cell exhaustion, thus potentially limiting the graft-versus-tumor effect.

#### Immuno-PET Targeting T-Cell Activation Markers

Targeting T-cell–restricted markers upregulated specifically during T-cell activation has the potential advantage of providing not only quantitative and qualitative information but also functional information about the activation status of T cells and their dynamics. Several approaches for different activation markers have been assessed preclinically.

HLA-DR is a human class II major histocompatibility complex molecule expressed on a variety of immune cells, including T cells during activation. A ^64^Cu (half-life, 12.7 h)-radiolabeled variable fragment of heavy chain antibodies was developed to target human HLA-DR and used to image T-cell activation in a murine model of xenogeneic GvHD (11). [^64^Cu]Cu-NOTA-HLA-DRVHH uptake was higher within the liver of mice displaying signs of severe GvHD (Fig. 2, middle panel) than in control mice, but the authors were unable to correlate early PET findings with subsequent GvHD before the occurrence of overt disease. Given the broad and unspecific expression of HLA-DR, the tracer uptake could be a consequence of tissue infiltration by activated T cells or by other non-T HLA-DR–positive cells, such as monocytes and macrophages.

OX40 is a member of the tumor necrosis factor receptor superfamily, and its cell-surface expression is highly restricted to activated T cells, on which it acts as a costimulatory molecule. We have previously developed a murine OX40-specific mAb ([^64^Cu]Cu-DOTA-OX40mAb) that enables noninvasive imaging of murine OX40-positive activated T cells (12). This tracer was assessed in vivo using a major histocompatibility complex–mismatch HSCT murine model of GvHD (13), given the increased expression and the role of OX40 during acute GvHD (14*,*^15^). OX40 immuno-PET successfully detected T-cell activation, expansion, and target-tissue infiltration. Importantly, because of its high sensitivity, OX40 immuno-PET could detect signs of GvHD even before the manifestation of clinical symptoms and could distinguish these signs from the toxicities of the conditioning regimen. However, a major limitation of this approach was the agonistic nature of the mAb used: at the mass doses used for PET imaging, the [^64^Cu]Cu-DOTA-OX40mAb tracer led to further T-cell activation and subsequent exacerbation of GvHD when administered early after HSCT. These results stress the need to develop biologically inert immuno-PET tracers for imaging purposes and to carefully select imaging targets and epitopes to avoid interfering with T-cell activation and disease pathogenesis.

A search for alternative target molecules for immuno-PET tracers to circumvent the toxicity encountered with OX40 immuno-PET identified the inducible T-cell costimulator (ICOS), an extracellular T-cell activation marker and costimulatory molecule, which is selectively upregulated on activated T cells during GvHD (16). Using a previously reported [^89^Zr]Zr-DFO-ICOSmAb tracer (17*,*^18^), we demonstrated that ICOS immuno-PET efficiently allowed monitoring of alloreactive T-cell activation, expansion, and tissue infiltration in a major histocompatibility complex–mismatch murine model of acute GvHD (16) (Fig. 2, right panel). Importantly, ICOS immuno-PET was not associated with any detectable toxicity and did not interfere with the graft-versus-tumor effect. The combination of highly specific and sensitive detection of T-cell activation, in the absence of detectable toxicity, renders ICOS immuno-PET a compelling method that warrants further evaluation in patients for the early detection of GvHD.

## IMAGING TISSUE DAMAGE

Once considered a passive target of T-cell cytotoxic function during acute GvHD, the target tissue epithelium is increasingly being recognized as an active player of GvHD pathogenesis. For this reason, target tissue damage can be exploited as a molecular imaging target to visualize phase 3 of the disease pathogenesis (Fig. 1). The analysis of phenotypic and functional changes of enterocytes during acute GvHD led to the identification of tryptophan-rich sensory protein (TSPO), a stress-related protein, as a marker expressed by enterocytes during acute GI-GvHD (19). TSPO is an outer mitochondrial membrane protein previously reported to be overexpressed by enterocytes after stimulation with inflammatory mediators such as tumor necrosis factor, leading to its overexpression in inflammatory bowel disease. After demonstrating enterocytic TSPO expression in tissue biopsies from patients with acute GI-GvHD, Scott et al. (19) performed a prospective pilot study of PET/CT using [^18^F]-flutriciclamide ([^18^F]GE-180), an already-reported third-generation high-affinity TSPO radiotracer, in 8 allogeneic HSCT adult recipients with a clinical suspicion of acute GI-GvHD. They demonstrated tracer uptake specifically at the intestinal level and correlation between uptake and histology in 6 of 8 participants (75%) including 4 true-positive and 2 true-negative. The correlation with histology was greater in small bowel and colon. Even though TSPO detection is not completely specific to enterocytes and although preliminary, this proof-of-concept study provided the first evidence for molecular imaging of target epithelium during GvHD as a strategy to detect tissue damage and potentially to monitor response to treatment during the healing process. Another drawback of [^18^F]GE-180 and other TSPO tracers is their sensitivity to the TSPO single-nucleotide polymorphism (rs6971-SNP), which affects the binding of these tracers. TSPO PET studies typically require patients to be genotyped to ascertain whether they are low-, medium-, or high-affinity binders and thus their eligibility for the scan.

## OPPORTUNITIES AND CHALLENGES FOR CLINICAL TRANSLATION

### Tracer Development and Optimization

The studies performed so far using the widely available PET tracer [^18^F]FDG have highlighted both the potential and the limitations of using molecular imaging for GvHD diagnosis, stressing the importance of developing new imaging agents designed with acute GvHD pathogenesis in mind. The ideal tracer will target molecules or biologic pathways specifically involved in acute GvHD, allowing for accurate distinction between GvHD-target tissues both from healthy tissues and from tissues affected by other pathologic processes, namely infections. Despite the well-established utility of murine models of GvHD for reproducing human GvHD pathogenesis, animal models are ultimately limited in their ability to fully assess the specificity of a tracer given the absence of confounding factors, such as infection risk, encountered in the clinic. It will therefore be essential to test the most promising tracer candidates in well-conducted clinical trials.

In addition to the target molecule/pathway recognized by the new tracers, the choice of PET radionuclide is also of crucial importance. Use of radionuclides with a longer half-life, such as ^89^Zr, might provide the advantage of obtaining sequential longitudinal images over several days, thus allowing imaging for both diagnosis and monitoring. The resulting prolonged radiation exposure and associated excretion in biologic materials may require specific radiosafety measures that may not be compatible with an outpatient setting. As such, shorter-lived radionuclides, such as ^64^Cu, also warrant consideration, as do advanced scanner technologies such as total-body PET, which are capable of generating high-resolution images with significantly reduced administered radioactive doses (20). Finally, combining molecular imaging by PET with anatomic modalities other than CT, such MRI, might further increase the diagnostic potential of new tracers, in particular immuno-PET tracers, similarly to what has been shown for [^18^F]FDG (5).

### Defining the Optimal Use of Molecular Imaging for GvHD Diagnosis and Monitoring

Once one or more promising radiotracers are identified, it remains to be defined how best to implement molecular imaging of GvHD into clinical practice. One option would be to use it as a screening strategy in all allogeneic HSCT recipients at selected time points after transplantation. Given the high complexity and significant costs of PET, it is, however, unlikely that this approach will prove to be cost-effective when used as a general screening tool. One option could be to restrict its use to patient populations at particular risk of developing GvHD on the basis of certain clinical criteria (e.g., the donor type used). Alternatively, the use of PET for detection of GI-GvHD could be triggered by other clinical signs frequently preceding or accompanying it, such as skin GvHD. Given the great results accomplished with blood biomarkers of GvHD, such as regenerating islet-derived 3α and suppression of tumorigenicity 2, we can imagine a scenario in which molecular imaging use will be triggered by positive results from the cheaper and more easily accessible blood biomarkers.

Virtually all patients receive GvHD prophylaxis after allogeneic HSCT in clinical practice. Early detection of GvHD using molecular therapy will likely not eliminate the need for such pharmacologic prophylaxis but could crucially help clinicians define its optimal duration and tapering schedule. Moreover, molecular imaging might indicate the transition from GvHD prophylaxis to therapy based on imaging findings and without the need to wait for clinical signs, and therefore tissue damage, before intervention, thereby enabling a personalized approach to GvHD treatment. Molecular imaging might be used to guide histologic sampling for GI-GvHD diagnosis in cases of clinical suspicion, thus increasing the sensitivity of tissue biopsies. In addition to acute GvHD diagnosis, molecular imaging has great potential as a tool to assess the severity of GvHD and guide therapy accordingly. We have recently seen promising results from clinical trials adjusting the intensity of anti-GvHD therapy on the basis of the severity of GvHD assessed on clinical and biologic criteria (21*,*^22^). Similarly, molecular imaging could be used to evaluate the extent and severity of GvHD in a more comprehensive way than is possible with endoscopy and biopsies, thus allowing identification of patients at low and high risk of GvHD and helping to adapt treatment protocols accordingly. Moreover, early reassessment using molecular imaging after treatment introduction might predict the response to therapy earlier than with currently available methods, thus allowing earlier adaptation of therapy.

## CONCLUSION

Diagnosis of acute GvHD currently relies on a combination of clinical symptoms and tissue biopsies. However, especially for liver and GI-GvHD, endoscopic biopsies are associated with significant morbidity and even mortality. Molecular imaging of GvHD has the potential to diagnose and monitor the disease while circumventing the use of invasive biopsies and to make a diagnosis earlier during GVHD, when intervention may be more successful. We have summarized how different molecular imaging strategies can be applied to the study of different phases of GvHD pathogenesis (Fig. 1), including [^18^F]FDG as a nonspecific but sensitive and versatile marker across all phases and several investigational target molecules for various molecular processes. Preclinical studies suggest that molecular imaging has the potential to detect the GvHD process before tissue damage and symptoms actually occur. Clinical trials are needed to define the optimal timing of molecular imaging for early GvHD diagnosis and additionally assess its potential for risk stratification and for monitoring response to therapy.

## DISCLOSURE

Israt Alam was supported by NIH/NCI (R01 CA286998-01). Federico Simonetta was supported by the Geneva Cancer League (LGC 20 11) and the Dubois-Ferriere-Dinu-Lipatti Foundation. No other potential conflict of interest relevant to this article was reported.
